# LIFE EVENTS AND CARDIOVASCULAR DISEASE EVENTS: THE STUDY OF WOMEN'S HEALTH ACROSS THE NATION

**DOI:** 10.1093/geroni/igac059.689

**Published:** 2022-12-20

**Authors:** Rachel Koffer, Rebecca Thurston, Karen Matthews

**Affiliations:** Arizona State University, Phoenix, Arizona, United States; University of Pittsburgh, Pittsburgh, Pennsylvania, United States; University of Pittsburgh, Pittsburgh, Pennsylvania, United States

## Abstract

Cardiovascular disease (CVD) is the number one cause of death for women, and major life events across midlife may contribute to CVD risk. The present study aimed to test whether greater exposure to major life events across nearly two decades of longitudinal follow-up would be associated with higher risk of clinical cardiovascular disease events. 3,222 middle-aged women from the multi-ethnic Study of Women’s Health Across the Nation reported and provided up to 15 years of major life events, non-fatal incident CVD events, traditional biobehavioral and sociodemographic factors, and death certificates. Cox proportional hazards models were used to test the association between average annual life events and incident fatal and nonfatal CVD events. Each additional major life event was associated with a 1.16-fold (95% CI: 1.08-1.23) increase in CVD events. CVD risk will be discussed considering evidence of racial/ethnic disparities in exposure to major life events.

